# Serum copeptin levels in adolescents with primary hypertension

**DOI:** 10.1007/s00467-013-2683-5

**Published:** 2013-12-29

**Authors:** Edyta Tenderenda-Banasiuk, Anna Wasilewska, Renata Filonowicz, Urszula Jakubowska, Marlena Waszkiewicz-Stojda

**Affiliations:** 1 Department of Pediatrics and Nephrology, Medical University of Bialystok, 17 Waszyngton Street, 15-274 Bialystok, Poland; 2 Emergency Department, Medical University of Bialystok, Bialystok, Poland

**Keywords:** Adolescents, Copeptin, Hypertension

## Abstract

**Background:**

The prevalence of hypertension continues to rise in the pediatric population. In recent years, there has been an increasing amount of reports on serum arginine vasopressin and its derivative, copeptin, in blood pressure control, but its role is still unclear. The objective of this study was to assess serum copeptin in adolescents with essential hypertension.

**Methods:**

The study cohort consisted of 84 subjects (30 girls and 54 boys) aged 11–18 years, divided into two groups: hypertension (HT) – 53 subjects with confirmed primary hypertension and R - reference group – 31 subjects in whom hypertension was excluded on the basis of ambulatory blood pressure monitoring (ABPM) (white-coat hypertension). Serum copeptin concentration was measured using a commercially available enzyme-linked immunosorbent assay kit (USCN).

**Results:**

Hypertensive patients had higher serum copeptin levels (median, 267 [Q1–Q3: 151.1–499.7 pg/ml]) than controls (median, 107.3 [Q1–Q3: 36.7–203.4 pg/ml]), (*p* < 0.01). Statistically significant difference was found both in males and females. In both groups, positive correlations between serum copeptin and uric acid levels (*r* = 0.31, *p* < 0.01), albuminuria (*r* = 0.45, *p* < 0.01), serum triglycerides (*r* = 0.3, *p* < 0.05), body mass index (BMI) standard deviation score (SDS) (*r* = 0.24, *p* < 0.05) and 24-h systolic blood pressure (SBP) (*r* = 0.37, *p* < 0.01) and diastolic blood pressure (DBP) (*r* = 0.23, *p* < 0.05) were found.

**Conclusions:**

In summary, higher serum copeptin levels, a surrogate for arginine vasopressin (AVP) release, are associated not only with systolic and diastolic blood pressure but also with several components of metabolic syndrome including obesity, elevated concentration of triglycerides, albuminuria, and serum uric acid level. However, for the time being, more research is needed in order to confirm the role of serum copeptin as a novel marker of elevated blood pressure and predictor of metabolic syndrome.

## Introduction

Pediatric hypertension is a lifelong problem that can be progressive from childhood into adulthood. Recent epidemiologic data indicate that the prevalence of hypertension has increased. In a representative sample of the US pediatric population aged 8 to 17 years, Rosner et al. [1] observed an increase in the prevalence of elevated blood pressure (BP) from National Health and Nutrition Examination Survey (NHANES) III to NHANES 1999–2008, with identical BP protocols. In this period, the risk for having elevated BP, both prehypertension and hypertension, increased by 27 % [1]. Previous reports by Ostchega et al. [2] who compared the prevalence of prehypertension in hypertension between NHANES III and each of NHANES 1999–2002 and 2003–2006 also confirmed this trend. The most important reasons are probably increased prevalence of obesity and higher salt intake in children and adolescents [1, 3].

The first studies on the role of arginine vasopressin (AVP) in controlling blood pressure were published in the year 1934 [4]. Since this time, there has been a large volume of published studies describing its role in hypertension, however the results have been equivocal. Experimental studies revealed that AVP antagonism may reduce arterial pressure in Goldblatt 1 and 2 kidney hypertension [5]. Plasma arginine vasopressin was reduced in primary aldosteronism, but elevated in malignant hypertension [5]. In essential hypertension, there was considerable disagreement among various studies in which plasma vasopressin or urine vasopressin excretion were measured as to whether there is evidence for increased secretion of vasopressin. Krakoff et al. [5] did not find any conclusive evidence that elevated AVP secretion occurs or is necessary for any form of clinical hypertension. Similarly, Kawano et al. [6] demonstrated that AVP did not play an important role in mild essential hypertension through its action on the V_1_ receptors regardless of dietary sodium intake. On the contrary, Padfield et al. [7] demonstrated that abnormal arginine vasopressin concentrations might be caused by hypertension. AVP has also been implicated as a significant contributor to blood pressure control in African Americans [8]. Similarly, AVP-dependent changes were found in elderly people [9], patients with congenital heart failure [10], or chronic kidney disease [11]. Blood pressure is regulated by many vasoactive factors. Among them, AVP is one of the most potent vasoconstrictors and is known to affect blood pressure by regulating vascular tone and body fluid through V1aR and/or V2R [12]. It was demonstrated that the renal vasculature and total renal blood flow are relatively insensitive to the action of vasopressin on the V_1a_ receptor under physiological conditions, but in pathological conditions, a renal vasoconstrictor response is observed. This year, an interesting paper on a vasopressin-dependent mechanism of hypertension was published by Littlejohn et al. [13]. The authors suggested that angiotensin-sensitive vasopressin-producing brain structures are major cardiovascular regulatory centers, especially in low-renin hypertension. The lack of consensus on the role of vasopressin in essential hypertension may be the result of the fact that AVP is an unstable molecule both in vivo and ex vivo. It is rapidly cleared from plasma and is largely attached to platelets in the circulation. Most vasopressin assays have relatively limited sensitivity. That is why in recent years an assay has been developed to measure plasma/serum copeptin, the C-terminal portion of the precursor of AVP. Because copeptin has a long half-life and is not bound to platelets, it is found in considerably higher concentrations in plasma than AVP and is easily detected with a validated sandwich assay. It is considered to be a reliable and clinically useful surrogate marker for AVP [14]. Copeptin levels correlate to AVP levels in plasma [15].

In recent years, there has been an increasing amount of literature on the role of plasma copeptin in metabolic syndrome [16], obesity [17], and progression of chronic kidney disease [18, 19]. However, to the best of our knowledge, serum copeptin has not been assessed in adolescents with essential hypertension.

The aim of this study was to test the hypothesis that serum copeptin (a surrogate marker of AVP) is increased in adolescents with essential hypertension. Additionally, we aimed to investigate the correlation between serum copeptin and clinical, laboratory and ambulatory blood pressure monitoring variables in this group of patients.

## Methods

This was a prospective cohort study of hypertensive adolescents. The study included 84 subjects (30 girls and 54 boys), aged 11–18 years, who were appointed to our unit (Department of Pediatrics and Nephrology, The Medical University of Bialystok, Poland) between May 2010 and September 2011 in order to confirm or rule out hypertension. The majority of subjects were referred to our department by general practitioners after finding elevated casual BP in a primary care office. On the basis of ambulatory blood pressure monitoring (ABPM), the examined adolescents were divided into two groups: hypertension (HT) – 53 subjects with confirmed primary hypertension and R - reference group – 31 subjects with white-coat hypertension.

Inclusion criteria were the following: an age of 11–18 years, primary arterial hypertension (verified by ABPM - mean 24-h systolic blood pressure levels greater than or equal to the 95th percentile for sex and height and systolic blood pressure (SBP) load greater than 25 %) [20], no clinical or laboratory signs of infection, normal levels of cortisol, thyroid-stimulating hormone (TSH) and normal renal function, no proteinuria, normal electrolyte levels, not having been treated with antibiotics within the last 4 weeks, and having signed informed-consent forms.

The following exclusion criteria were used: heart failure, renal or hepatic dysfunction, diabetes mellitus, systemic inflammatory conditions, autoimmune diseases, subjects with clinical or laboratory signs of secondary hypertension (documented thyroid, kidney or heart disease, abnormal Doppler of renal arteries), girls treated with contraceptive pills, patients treated with hypertensive agents, and medications known to affect serum uric acid levels and blood pressure values.

The reference group consisted of 31 subjects with normal blood pressure confirmed in ABPM (mean 24-h systolic blood pressure levels less than the 95th percentile for sex and height and SBP load less than 25 %). The participants were term-born, with normal birth weight, and did not receive any medication at the time of the examination. Family history of adolescents qualified for this group did not reveal hypertension or other cardiovascular diseases. History of diabetes or gout was negative as well. Blood and urine testing including serum creatinine, urea, serum fasting glucose, lipid profile, and serum uric acid concentration was within the normal range.

The protocol was approved by the Bioethics Committee of The Medical University of Bialystok in accordance with the Declaration of Helsinki. Informed consent was obtained from parents of all participants and children older than 16 years of age.

For all subjects, careful clinical histories were taken and physical examinations were performed. Body weight and height were measured using a balance beam scale and pediatric wall-mounted stadiometer and body mass index (BMI) was calculated as weight (in kilograms) divided by the square of height (meters squared). Age- and height-specific reference values for BMI and height were generated by the LMS method [21], which characterizes the distribution of a variable by its median (M), the coefficient of variation (S, i.e., the ratio of the SD and mean), and skewness (L) required to transform the data to normality. The LMS values were taken from the OLAF study, published by Kulaga et al. [22]. Similarly, because the use of pediatric ABPM reference values is comprised by the non-Gaussian distribution of 24-h blood pressure in children, we used the LMS method to calculate SDS values for ABPM. The reference values of* L*,* M*, and *S* interpolated for the child’s height from those of the published reference population of healthy German children [23].

After an overnight fasting, 5 ml of venous peripheral blood was collected from each patient and reference for routine laboratory testing. Isolated serum aliquots were stored at −80 °C for further analysis. The biochemical work-up included serum creatinine, urea, serum fasting glucose, lipid profile, and serum uric acid concentration.

The serum concentration of copeptin was measured using a commercially available enzyme-linked immunosorbent assay (ELISA) kit (USCN) according to the manufacturer’s instructions. Serum copeptin levels were expressed in pg/ml.

Serum creatinine was determined by Jaffe reaction and uric acid was assessed using the colorimetric method, both on the Hitachi apparatus. Serum cholesterol, HDL cholesterol, and triglycerides were determined by the enzymatic method using Hitachi 912 Chemistry Analyzer (La Roche Company, Tokyo, Japan). Serum glucose was measured with the Integra 800 analyzer. Glomerular filtration rate was assessed updated Schwartz’s formula (GFR), which is recommended in pediatric population [24]. The 24-h urinary albumin excretion rate (UAER) was analyzed by radioimmunoassay method (RIA). Albuminuria was considered as 24-h UAER >30 mg/24 h. Micro- and macroalbuminuria were defined as 24-h UAER values of 30–300 mg/24 h and >300 mg/24 h, respectively.

Ambulatory blood pressure monitoring (ABPM) was performed by the oscillometric method using Spacelabs Medical Model 90207 apparatus (Spacelabs Inc, Richmond, Washington, USA).

The monitors were programmed to measure BP every 15 min during daytime (8:00–22:00) and every 30 min during nighttime (22:00–8:00), however, the periods were corrected according to the patients’ diaries. Recording started between 8:00 and 9:00 a.m. and lasted for 24 h. Recordings with a minimum 80 % of measurement and without break longer than 2 h were considered sufficient. The mean SBP and diastolic blood pressure (DBP) were calculated separately for the 24-h period and for awake and asleep periods. Additionally, systolic blood pressure load (SBPL) and diastolic (DBPL) blood pressure load during the day and night was calculated. HT on the basis of ABPM was defined as mean systolic BP levels that are ≥95th percentile and LSBP more than 25 % [20]. Data analysis was performed using the computer program Statistica 10.0 PL (StatSoft, Tulsa, USA). Discrete variables were expressed as counts (percentage), whereas continuous variables as median and quartiles, unless stated otherwise. The comparison between the two groups was done with the Chi-square and Fisher’s exact tests for categorical variables and *t* test for continuous variables for normally distributed data or Mann–Whitney test for the data distributed not normally. Correlations between copeptin and other variables (clinical and laboratory parameters) were evaluated by Pearson’s or Spearman’s test as appropriate in both groups. A value of *p* < 0.05 was considered statistically significant.

## Results

The demographic, clinical, and laboratory data for each group are summarized in Table 1. Ambulatory blood pressure monitoring and laboratory results were successfully collected from 84 adolescents, 53 hypertensive and 31 with white-coat hypertension (reference group). The median age did not differ between the groups. Males were more frequently affected with HT than females, consistent with the available reports in the area [25]. In the examined group, 45 subjects (84.9 %) were males (M) and eight (15.1 %) were females (F), whereas more girls (*n* = 22, 71.0 %) than boys (*n* = 9, 29.0 %) were found in the reference group.

**Table 1:** Anthropometric, clinical, and metabolic characteristics of the examined group (HT) and the reference group (R)

	R (31)	HT (53)	*p*
Sex (M/F)	9/22	45/8	<0.01
Age (years)	16 (15–17)	17 (16–17)	NS
Height SDS	0.04 (−0.95–1.25)	0.33 (−0.54–0.91)	NS
BMI SDS	0.00 (−0.44–1.25)	1.32 (0.60–2.36)	<0.01
24 SBP SDS	0.10 (−0.88–0.89)	1.68 (0.67–2.62)	<0.01
24 DBP SDS	0.21 (−0.53–0.78)	1.17 (0.57–1.79)	<0.05
CRP (mg/dl)	0 (0–0.1)	0 (0–0.1)	NS
GFR ml/min/1,73 m^2^	157.4 (143.2–187.9)	145.6 (129.8–155.9)	<0.01
Uric acid (mg/dl)	4.58 (3.42–4.84)	6.49 (5.83–7.07)	<0.01
Cholesterol (mg/dl)	166.5 (147.0–183.0)	162.0 (139.0–189.0)	NS
HDL cholesterol (mg/dl)	52.5 (49.0–60.0)	54.0 (44.0–58.0)	NS
Triglyceride (mg/dl)	78 (64–91)	104 (73–163)	<0.05
Triglyceride/HDL cholesterol	1.63 (1.22–1.88)	2.00 (1.33–3.45)	NS
Glucose (mg/dl)	89 (86–91)	92 (89–95)	NS
Albuminuria (mg/24 h)	4.35 (3.12–6.72)	6.13 (3.57–9.3)	NS
Copeptin (pg/ml)	107.3 (36.7–203.4)	267 (151.1–499.7)	<0.01

*BMI* body mass index, *24 SBP* 24-h systolic blood pressure, *24 DBP* 24-h diastolic blood pressure, *CRP* C-reactive protein, *GFR* glomerular filtration rate, *SDS* standard deviation score, *HDL* high-density lipoprotein

Values are presented as the median with the interquartile range (Q_1_–Q_3_)

The body height SDS was similar in both groups, however BMI SDS was significantly higher in hypertensive patients (*p* < 0.01).

In the HT group, there were 43 patients with hyperuricemia (serum uric acid level ≥5.5 mg/dl and ten with normouricemia).

Serum levels of fasting glucose, total cholesterol and HDL, and LDL cholesterol in studied patients did not differ from the reference group (*p* > 0.05). Serum triglyceride levels were significantly higher in the HT group (median, 104 mg/dl) than in the reference group (78 mg/dl)(*p* < 0.05).

**Fig. 1:**
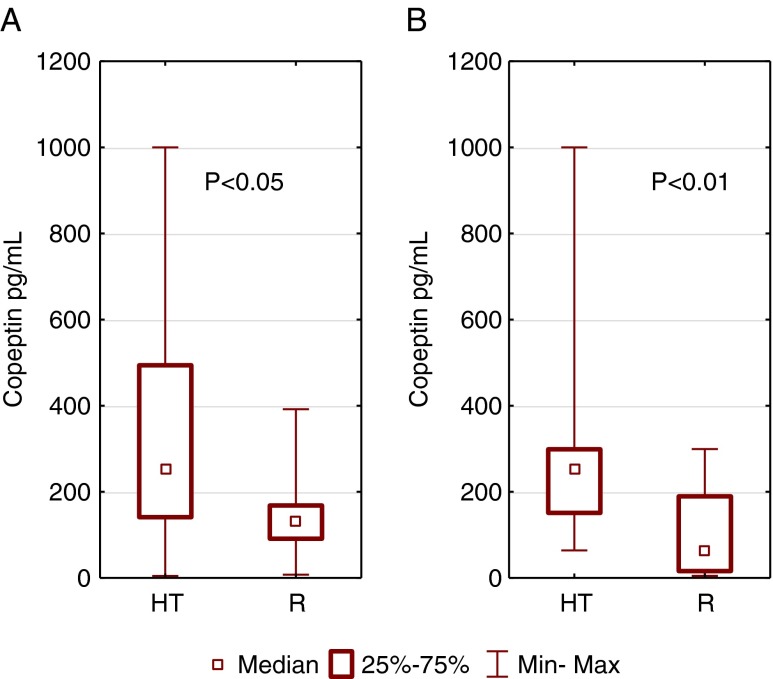
Comparison of serum copeptin in males (**a**) and females (**b**) between hypertensive (HT) and reference (R) group

When compared to the reference group, hypertensive patients had higher serum copeptin levels (Table 1). Figure 1 presents a comparison between serum copeptin in the hypertensive and reference groups separately for males and females. In hypertensive males, the serum copeptin level was significantly higher when compared to reference group (median, 269.5 pg/ml [Q1–Q3: 150.1–499.6 pg/ml]) vs. reference (median, 153.3 [Q1–Q3: 91.0–223.7 pg/ml])(*p* < 0.05). A similar trend was observed in females. The serum copeptin level in HT females was a median of 251.9 [Q1–Q3: 151.1–298.7 pg/ml]) vs. controls (median, 76.68 [Q1–Q3: 15.85–197.9 pg/ml]) and the difference was statistically significant (*p* < 0.01).

In both groups, serum copeptin was significantly higher in males than in females (*p* < 0.05). A statistically significant positive correlation was observed between serum copeptin and uric acid levels (*r* = 0.31, *p* < 0.01), albuminuria (*r* = 0.45, *p* < 0.01), serum triglycerides (*r* = 0.3, *p* < 0.05) and atherogenic ratio (serum triglyceride/HDL cholesterol ratio) (*r* = 0.33, *p* < 0.05), BMI SDS (*r* = 0.24, *p* < 0.05).

Additionally, a significant positive correlation was also found between serum copeptin and 24-h SBP SDS (*r* = 0.37, *p* < 0.01) and 24-h DBP (*r* = 0.23, *p* < 0.05) and nighttime BP load (SBPL, *r* = 0.37, *p* < 0.01; DBPL, *r* = 0.26, *p* < 0.05) (Table 2).

**Table 2:** Correlations with serum copeptin in all examined teenagers

	Correlation coefficient (*r*)	Significance level (*p*)
Age (years)	−0.01	NS
BMI Z-score	0.24	<0.05
Serum uric acid (mg/dl)	0.31	<0.01
Serum triglyceride (mg/dl)	0.30	<0.05
Triglyceride/HDL cholesterol ratio	0.32	<0.05
Albuminuria (mg/24 h)	0.45	<0.01
GFR ml/min/1.73 m^2^	−0.18	NS
24 SBP SDS	0.37	<0.01
24 DBP SDS	0.23	<0.05
Night SBPL (%)	0.37	<0.01
Night DBPL (%)	0.26	<0.05

*BMI* body mass index, *GFR* glomerular filtration rate, *24 SBP* 24-h systolic blood pressure, *24 DBP* 24-h diastolic blood pressure, *SBPL* systolic blood pressure load, *DBPL* diastolic blood pressure load, *SDS* standard deviation score, *HDL* high-density lipoprotein

The factors that were found to have a significant correlation with serum copeptin in the single regression analyses were used as explanatory variables to create the multiple regression models. In the model (Table 3), three parameters (serum uric acid, systolic BP, and BMI Z-score) accounted for more than 38.8 % of the variation in the serum copeptin level (*p* < 0.01).

**Table 3:** Multiple linear regression analysis of the serum copeptin level

Variables	Coefficient	Standard error	95 % confidence interval	*p* value
Uric acid (mg/dl)	−130.285	56.4930	(−243.16; −17.39)	0.03
Systolic blood pressure	7.759	3.6551	(0.46; 15.05)	0.03
BMI Z-score	39.332	16.2896	(6.79; 66.50)	0.01

*BMI* body mass index

## Discussion

The most interesting finding of this cross-sectional study was that serum copeptin concentration, a surrogate for plasma AVP release, was significantly higher in hypertensive adolescents in comparison to teenagers in whom diagnosis of hypertension was not confirmed in 24-h ambulatory blood pressure monitoring (reference group – white-coat hypertension). It is interesting to note that in both the hypertensive and reference groups serum copeptin levels were higher than those reported in the literature in healthy children [26]. A possible explanation for this might be that different methods are used to measure plasma or serum copeptin level and the results are given in different units. However, it seems possible that these results are due to the fact that the reference group in our study included not healthy teenagers but adolescents with white-coat hypertension, in whom serum copeptin might be increased. Further studies on a representative group of children is needed to find reference values for age and gender.

Because there is a significant difference in gender distribution between patient and reference group, we analyzed serum copeptin in gender subgroups separately and confirmed increased values both in hypertensive males and females when compared to reference subgroups. Serum copeptin correlated positively with both 24-h systolic and diastolic blood pressure SDS and nighttime blood pressure load. However, no correlations with nocturnal dip in hypertensive patients were observed. To the best of our knowledge, there is no data in the literature on serum copeptin levels and its correlation with ambulatory blood pressure monitoring. Another important finding of this study was that serum copeptin levels were significantly higher in males than in females. This observation is consistent with that of Enhorning et al. who found higher levels of plasma copeptin in men than in women in an adult population [17]. In an experimental study, AVP secretion was similar in both male and female mice, however sex-specific differences in the rate of AVP degradation/clearance were observed.

Several lines of evidence support a role for AVP in the pathogenesis of hypertension. Activity of the local tissue RAS within the brain has been implicated in the development of hypertension. It was documented in experimental studies that brain RAS modulates the cardiovascular and fluid-electrolyte homeostasis not only by interacting with the autonomic nervous system but also by modulating the hypothalamic–pituitary axis and vasopressin release [27].

Two major mechanisms have been proposed that account for the blood pressure effects of brain angiotensin. First, action of the RAS within the supraoptic and paraventricular hypothalamic nuclei stimulates the production and release of arginine vasopressin [28–30]. Second, brain stem actions of RAS alter baroreflex function sympathetic output [31, 32]. Surprisingly, a population of AVP-expressing neurons appeared to be involved in the regulation of sympathetic nervous activity. It can thus be suggested that AVP-mediated cross-talk between these two mechanisms exists.

There are three receptor subtypes known that can bind vasopressin, theV_1a_, V_1b_, and V_2_ receptors. The V_1a_ and V_2_ receptors mediate a number of different cellular effects leading to water conservation. The V_1a_ receptor is localized in smooth muscle cells, the liver, and kidney (mainly in the interlobular arteries, the descending vasa recta, the macula densa, and the collecting duct) [33]. Activation of the V_1a_ receptor results in an increase in blood pressure by vasoconstriction. This increased vasoconstriction is the result of a direct effect on smooth muscle cells and of an indirect effect caused by increased renin secretion [34]. It has also been suggested that VP may induce HT by V_2_ receptor-mediated increased tubular sodium retention [35, 36]. Additionally, a complex interplay between V_1a_ − and V_2_− receptor-mediated effects has been suggested [35, 37]. Experimental studies showed that chronic blockade of vasopressin V_1a_/V_2_ receptor resulted in normalization of blood pressure in mice [12].

Other important findings were positive correlations between serum copeptin and body weight, BMI SDS, serum uric acid, triglycerides levels, TG/HDL ratio (atherogenic index), and albuminuria.

Association of plasma copeptin and abdominal obesity was confirmed by Enhorning et al. in adults [16]. It is interesting to note that authors demonstrated that elevated copeptin predicts the development abdominal obesity, microalbuminuria and even diabetes mellitus during long term follow up [16]. The role of AVP in pathogenesis of both diabetes mellitus and abdominal obesity was described before; however, it is still not well known. The results of Enhorning et al.’s study suggested that AVP triggers two different pathways leading to DM and abdominal obesity. Independently, however, this fact should be confirmed in further studies. In this study, we found a strong positive correlation between serum copeptin and BMI SDS, however we did not measure waist circumference in our patients. Serum glucose levels in our patients were normal and did not differ with the reference group. No correlation between serum copeptin and serum glucose levels was found. This may be caused by the early stage of metabolic syndrome in our patients. The contribution of AVP to glucose and lipid metabolism is rather complex. AVP mediates gluconeogenesis and glycogenolysis through V_1a_ receptors in the liver [38, 39] and stimulates glucagon or insulin, depending on levels of glycemia. Additionally, AVP exerts an anti-lipolytic action [40]. In our cohort, mean serum cholesterol level did not differ when compared with the reference group, but serum TG levels were higher in hypertensive patients. Serum copeptin was significantly associated with higher triglyceride levels and lower HDL cholesterol levels. The findings of the current study are consistent with those of Saleem et al. [41] who found serum copeptin to be independently associated with several components of metabolic syndrome including adiposity and dyslipidemia (lower HDL cholesterol and higher triglyceride levels). In an experimental study, Rossi et al. [42] demonstrated that intraperitoneal injection of AVP in goats resulted in an increase in plasma triglyceride levels. One of the possible explanations for this correlation is increased hepatic synthesis of triglycerides under the influence of glucocorticoids, glucagon, and epinephrine released under stress, all of which are up-regulated by AVP [42].

In our study, although albuminuria was not significantly higher in the HT group, we found a positive correlation with serum copeptin. An association between these two parameters was also confirmed by Enhörning et al. [17], however in elderly people, it might be caused by AVP- mediated changes of glucose metabolism or blood pressure.

What is interesting is that we also found a statistically significant positive correlation between serum copeptin and serum uric acid. It is difficult to explain this result, but it might be indirectly related to hypertension, which is associated with hyperuricemia in this group of patients.

Several limitations of our study need to be mentioned. This study is limited by its small sample size and single-center patient population. The study is designed to correlate serum copeptin level values of blood pressure measured in ambulatory blood pressure monitoring. However, in the course of study, we noticed significant correlations with metabolic syndrome, but we were not able to relate copeptin to waist circumference and HOMA index.

In summary, higher serum copeptin levels, a surrogate for AVP release, are associated not only with systolic and diastolic blood pressure but also with several components of metabolic syndrome including obesity, elevated concentration of triglycerides, albuminuria, and serum uric acid level. However, for the time being, more research is needed in order to confirm the role of serum copeptin as a novel marker of elevated blood pressure and predictor of metabolic syndrome.
